# Assessing How Healthcare Providers Find Information Regarding the Benefits and Harms of Treatments in Response to Patient Inquiries at an Independent Academic Center: A Cross-Sectional Study

**DOI:** 10.7759/cureus.96297

**Published:** 2025-11-07

**Authors:** Rahul Mhaskar, Bryan G Kane, Robert Barraco

**Affiliations:** 1 Internal Medicine, Medical Education, University of South Florida Morsani College of Medicine, Tampa, USA; 2 Emergency and Hospital Medicine, University of South Florida Morsani College of Medicine, Lehigh Valley Health Network, Bethlehem, USA; 3 Trauma Surgery, Lehigh Valley Health Network, Allentown, USA

**Keywords:** google, shared decision-making, side effects, treatment benefits, up to date

## Abstract

Background

Patients with medical information have become active participants in their treatment process; however, this has created additional challenges for physicians and other healthcare providers. However, how providers cope with patients’ requests for such information is not well understood, and the information-seeking behavior of physicians in catering to patients’ information needs has not been thoroughly studied. The primary objectives of this cross-sectional study were to understand healthcare providers’ information-seeking behavior when responding to patients’ specific questions about the benefits and risks of treatments.

Methodology

This Institutional Review Board-approved study was conducted at an independent academic center in Allentown, PA, between 2017 and 2020. We collected pertinent data from structured one-on-one interviews using an interview guide. The interviews were recorded using an electronic audio recorder that saved the recordings as an audio file. Interview transcripts were analyzed using hand coding. We investigated the relationship between categorical participant attributes using the chi-square or Fisher’s exact test at a significance level of 0.05, with continuity correction. We used the Kruskal-Wallis and Mann-Whitney U tests to investigate the differences between the distribution of continuous variables across the participants’ categorical attributes.

Results

A total of 124 providers from eight departments participated. The majority, 62% (77/124) of the providers, reported that patients brought information about treatment, and 56% (69/124) about a diagnosis. We did not notice a significant variation in the number of patients who brought in information related to their diagnosis (p = 0.08), prognosis (p = 0.35), and other topics, such as birth control, food allergies, and vaccines (p = 0.13), across medical specialties. Overall, 72% (89/124) of the providers reported using DynaMed, UpToDate, and Lexicomp, and 54% (67/124) used PubMed. Further, 27% (33/124) referenced the clinical practice guidelines, 17% (21/124) of the providers referred to textbooks, and 15% (19/124) discussed the topic with colleagues. Moreover, 35% (44/124) of the providers reported conducting traditional critical appraisals to determine the credibility of the information. We did not observe a significant variation in the number of providers using the library website to access journals and the PubMed database (p = 0.29) or in the number of providers seeking information from their colleagues (p = 0.58) across medical specialties. Providers who have recently finished their training (median experience = 6 years; range = 3-15) reported being not satisfied with their process of seeking information compared with providers with a greater level of experience (median = 15 years; range = 3-45) reporting being satisfied and (median = 14.5 years; range = 4-34) reporting being somewhat satisfied (p = 0.04).

Conclusions

We found that most physicians across all medical specialties utilized and preferred point-of-care tools, such as DynaMed and UpToDate. However, many providers still rely on the reputation of the information source, such as a journal’s impact factor and the author’s research credentials, to determine the credibility and reliability of the information.

## Introduction

Patients often use internet-based resources to assess information about diseases and treatments. For example, 39% of cancer patients (~2.3 million) in the industrialized world have reported accessing the internet for cancer-related information [1]. Patients increasingly visit clinicians for information about their symptoms, diagnoses, and treatment options. Patients with medical information have become active participants in their treatment process; however, this has created additional challenges for physicians and other healthcare providers [2].

Many patients suffer from complex diseases for which multiple treatment options may exist, and continuous research progress constantly influences the treatment algorithm [3]. With limited time, providers must stay up-to-date on the latest treatment modalities and help patients distinguish between reliable and unreliable information [4]. As many diseases are associated with significant morbidity, stress, and costs, patients desire concrete answers from their providers regarding the benefits (e.g., survival, response rate, cure) and harms (treatment-related mortality and morbidity) of treatment(s). However, how providers cope with patients’ requests for such information is not comprehensively known. Oncology is one specialty where provider treatment decisions have explicitly been studied [5,6]. Outside of oncology, the more general information-seeking behavior of physicians and advanced practice clinicians (APCs) has been described [7-9].

We conducted a hypothesis-generating cross-sectional study of the entire medical staff. The goals of our study were to collect data on providers’ information-seeking behavior regarding the answers to patients’ specific questions related to the benefits and harms of treatments. The objective of this study is to describe the information-seeking behaviors of healthcare providers when addressing patient questions about the benefits and harms of treatment. Interim findings of this research were previously presented as a poster at the LVHN Research Scholar Program Poster Session, Lehigh Valley Health Network, Allentown, PA, in 2019 and 2020.

## Materials and methods

This Institutional Review Board (IRB)-approved study was conducted at an independent academic center in Allentown, PA, during 2017 and 2020. This center hosts 120 medical students, 234 residents, and 47 fellows. We included board-certified/board-eligible physicians or APCs, available for a face-to-face, 30- to 40-minute interview, who were full-time employees of the hospital and were willing to sign an informed consent form, which included permission to take both handwritten notes and be recorded on audio recording during the interview.

We employed stratified purposive and random purposive sampling techniques, which involved dividing the purposefully selected target population into strata (i.e., health specialties such as internal medicine, pediatrics, family medicine, dentistry, etc.), intending to discover elements that are similar or different across strata (e.g., information-seeking behavior similar or dissimilar across specific specialties) [10]. We employed stratified, purposive, and random sampling techniques to ensure a diverse sample from the entire medical staff. Recruitment involved sending emails with a study cover letter, distributing IRB-approved flyers at on-site meetings, or a personal approach by a study team member. Physicians and APCs consenting to participate contacted the study team and scheduled an interview. The interviewees informed the participants about the study context, including the hypotheses, at the beginning of the interview. The participants were not compensated in any manner.

As the interviewer was a critical component to the successful implementation of our study, the interviewers underwent intensive training. We conducted formal training sessions for the interviewers, addressing interviewing techniques, standardization of interviews, qualitative data collection, and data entry. Furthermore, the interviewers practiced and improved their skills by interviewing volunteer faculty. Faculty study sub-investigators observed these initial interviews and gave feedback to the trainees.

All interviews were conducted using the one-on-one format at a place convenient for the participating provider. Using an interview guide (Appendices), the study coordinator and other study team interviewers conducted qualitative, open-ended, in-depth interviews to minimize the imposition of predetermined responses when gathering data [10].

The interviews were recorded using an electronic audio recorder that saved the recordings as an audio file. Backup audio recorders were used to prevent data loss, and the data were transcribed independently by one of the researchers in a blinded fashion. This method of collecting qualitative interview data has been previously used and shown to be reliable [10].

All the data, including the audio files of the interview and the transcribed data, were kept under at least two locks and accessible only to the study staff.

Data analysis

Interview transcripts were analyzed using hand-coding. Content analysis via hand coding was conducted using an intuitive or immersion/crystallizing analysis plan. The researcher reviewed the data and identified the aspects most relevant to the objectives. Validity was determined through peer debriefing, whereby the entire research team reviewed, validated, and verified all interpretations and conclusions drawn from the data (consensual validity). Each interview transcript was reviewed as an individual unit, and responses were segmented by interview questions to allow for the extraction of themes and a comparison of participants. The transcribing and coding processes were conducted independently by two researchers. In the event of discrepancies, a consensus was reached by reviewing the audiotape with senior faculty members and discussing the findings. The independent review and triangulation of the data helped reduce coder bias.

We conducted descriptive analyses of the relevant variables. The mean and standard deviation, or the median and range, are reported for continuous variables. We report frequencies for categorical variables. We investigated the relationship between categorical participant attributes using the chi-square test or Fisher’s exact test at a significance level of 0.05 with continuity correction. We used the Kruskal-Wallis and Mann-Whitney U tests to investigate the differences between the distribution of continuous variables (for example, the average percentage of patients who bring questions to the consultation) across the participants’ categorical attributes. We did not use any imputation for missing data. All statistical analysis was conducted using SPSS version 29.0 software (IBM Corp., Armonk, NY, USA).

## Results

A total of 124 providers from eight departments, each with numerous divisions, participated in the study. The majority, 86% (107/124), had completed their undergraduate medical education in the United States. More than half, 57% (71/124) of our participants, were from cognitive specialties (internal medicine, family medicine, pediatrics, neurology, psychiatry, and cardiology). The median duration since completing undergraduate medical education was 21 years (range = 4-45 years), and the median duration since completing the fellowship was 15 years (range = 4-45 years). Due to the cross-sectional nature of the enrollment, there was variation in the number of patients seen per month (median = 200, range = 30-720). Similarly, there was variation in the percentage of patients (out of all patients seen by a healthcare provider in a month) who had searched for the consultation (median = 6.5, range = 0-90). The distribution of the number of patients seen per month by individual specialties was similar (p = 0.82). Similarly, the average number of patients bringing information they had searched for to the consultation was similar (p = 0.07) across medical specialties.

The majority, 62% (77/124) of the providers, reported that their patients brought information about treatment. In comparison, 56% (69/124) of the providers reported that their patients had brought diagnosis-related information to the consultation. We did not notice a significant variation in the number of patients who brought in information related to their diagnosis (p = 0.08), prognosis (p = 0.35), and other topics, such as birth control, food allergies, and vaccines (p = 0.13), across medical specialties. However, only 33% of emergency medicine providers reported that their patients brought information about the treatment, compared with providers from medical specialties (73%), surgical specialties (72%), and dentistry (60%) (p = 0.04) (Table 1).

**Table 1:** Participant characteristics by specialty. *: Values reported are median (minimum to maximum). **: Other topics included birth control, food allergies, and vaccines. #: P-values were calculated using the Mann-Whitney U test. All other p-values were calculated using the chi-square or Fisher’s exact test.

Participant characteristics	Specialty				P-value
	Emergency Medicine	Medicine	Surgery	Dentistry	
Patients who are seen per month*	200 (80–300)	200 (40–600)	220 (30–600)	200 (65–250)	0.822^#^
Patients bring the information they searched for to the consultation*	2 (0–15)	9 (1–76)	10 (1–90)	1 (1–5)	0.077^#^
Patients bringing information about					
Diagnosis	92	61	48	60	0.082
Treatment	33	73	72	60	0.048
other topics**	0	11	14	40	0.134
Prognosis	0	6	13	0	0.353
Treatment benefits	57	56	80	63	0.116
Side effects	50	55	33	40	0.252
Providers use the following to gather information					
Journals and PubMed	43	51	69	60	0.292
Their colleagues	21	14	21	0	0.587
Point-of-care tools	71	83	55	20	0.002
Clinical practice guideline	0	20	38	80	0.001
Textbooks	21	13	21	20	0.699
Provider’s assessment of evidence credibility using					
Traditional critical appraisal	31	43	33	40	0.756
Journal’s impact factor/reputation	54	61	63	100	0.327
Peer-reviewed publications	0	11	11	20	0.556
Citations review	31	5	7	0	0.015
UpToDate	8	3	4	0	0.839
Provider’s assessment of evidence reliability using					
Reputation of the source	42	41	62	25	0.388
Traditional critical appraisal	33	41	44	75	0.531
Peer-reviewed publications	25	20	19	25	0.975
Repetition/Reproducibility	0	26	12	0	0.107
Published evidence matches their clinical findings	8	8	12	0	0.874
Provider’s perceived ease of access to information	93	87	96	100	0.415

Almost all the patients (104/124) had searched the internet for information. In contrast, only a few (9/124) had relied exclusively on other sources, such as family, friends, television commercials, and print media. Most providers, if not all, reported acceptance and tended to motivate the patients. The majority also mentioned that they have had informed discussions with their patients, providing them with relevant information. Some providers (5/124) expressed frustration over the quality and reliability of the information brought by the patients.

Overall, 64% (79/124) of the providers reported that their patients had specific questions about treatment benefits and other treatment options, including relative costs, benefits, and other related concerns. Over half (65/124) of the providers reported that their patients had specific questions regarding the side effects of treatments. We did not observe a significant variation in the number of patients with treatment benefits (p = 0.11) and side effects-related questions (p = 0.25) across medical specialties (Table 1).

Further, 72% (89/124) of the providers reported using point-of-care tools such as DynaMed, UpToDate, and Lexicomp, and 54% (67/124) stated that they utilized the library website to access journals and the PubMed database for searching information regarding the benefits and harms of treatments. Moreover, 27% (33/124) of providers referenced the clinical practice guidelines from their individual specialties’ societies, 17% (21/124) of providers referred to textbooks, and 15% (19/124) of providers sought assistance from their colleagues for searching information regarding the benefits and harms of treatments.

We did not observe a statistically significant difference in the number of providers using the library website to access journals and the PubMed database (p = 0.29). Similarly, the number of providers across medical specialties seeking information from their colleagues was similar (p = 0.58) (Table 1). However, only 20% of dentists reported using point-of-care tools such as DynaMed, UpToDate, and Lexicomp, compared with providers from medical specialties (83%), surgical specialties (55%), and emergency medicine specialties (71%) (p = 0.002). On the other hand, 80% of dentists reported that they referenced the clinical practice guidelines compared with medical (20%), surgical (38%), and emergency medicine (0%) specialty providers (p = 0.001) (Table 1).

We also did not notice any statistically significant association between the providers’ experience and their use of Journals/PubMed (p = 0.54), clinical practice guidelines (p = 0.76), textbooks (p = 0.25), or assistance from colleagues (p = 0.68). However, providers with less experience were more likely to use the point-of-care tools than those with more experience (p < 0.001).

Further, 35% (44/124) of the providers reported conducting traditional critical appraisals to determine the credibility of information they seek regarding the benefits and harms of treatments. Similarly, 34% (42/124) reported that they value the reputation of the information source (journal or author) to determine its credibility. Moreover, 9% (11/124) of the providers reported that they considered peer-reviewed publications credible, and 7% (9/124) reviewed the references to view a source as credible.

We did not notice a statistically significant variation in the number of providers using traditional critical appraisal (p = 0.75), the reputation of the information source (p = 0.32), peer-reviewed publications (p = 0.55), and UpToDate (p = 0.83) to gauge the credibility of information sought to address benefits and harms of treatments across medical specialties (Table 1). However, 31% (4/13) of emergency medicine providers reported that they review references/citations to consider a source as credible, compared with medical (5%), surgical (7%), and dental (0%) specialty providers (p = 0.01) (Table 1).

We did not detect any statistically significant association between providers’ experience levels and their use of the journals’ impact factor/reputation (p = 0.83), UpToDate (p = 0.73), citation review (p = 0.85), peer-reviewed publications (p = 0.13), and the traditional critical appraisal to determine the credibility of information (p = 0.05) (Table 2).

**Table 2:** Providers’ experience level and their information-seeking behavior. *: Values indicate median (minimum-maximum) years of experience. **: P-values were calculated using the Mann-Whitney U test.

Participant characteristics	Yes*	No*	P-value**
Providers use the following to gather information			
Journals and PubMed	14 (3–43)	15 (3–34)	0.541
Their colleagues	14 (4–30)	14 (3–43)	0.687
Point-of-care tools	12 (3–34)	19.5 (4–43)	0.001
Clinical practice guidelines	13 (3–43)	15 (3–43)	0.762
Textbooks	15.5 (5–43)	13 (3–43)	0.252
Provider’s assessment of evidence credibility using			
Traditional critical appraisal	12 (3–43)	17 (3–43)	0.527
Journal’s impact factor/reputation	15 (3–43)	14 (3–43)	0.839
Peer-reviewed publications	19 (3–29)	13.5 (3–43)	0.136
Citations review	16 (4–34)	14.5 (3–43)	0.851
UpToDate	14 (4–22)	15 (3–43)	0.731
Provider’s assessment of evidence reliability using			
Reputation of the source	12.5 (3–43)	12 (3–36)	0.893
Traditional critical appraisal	14 (3–43)	12 (3–34)	0.435
Peer-reviewed publications	12 (3–34)	13 (3–43)	0.846
Repetition/Reproducibility	11 (3–29)	14 (3–43)	0.293
Published evidence matches their clinical findings	20 (3–28)	12 (3–43)	0.410

Further, 29% (36/124) of the providers reported that they rely on the reputation of the information source to determine its reliability. Similarly, 27% (34/124) said that they employ traditional critical appraisal to assess the reliability of the information. Moreover, 14% (18/124) of the providers reported that they find peer-reviewed publications reliable, and 12% (15/124) labeled the information as reliable if repeated or reproduced, that is, if numerous other authors reported similar findings. At the same time, 6% (7/124) of the providers reported that they find the information reliable if it matches their clinical results or real-world experience.

We did not notice a statistically significant variation across specialties in the providers’ approaches to using the reputation of a source (p = 0.38), traditional critical appraisal (p = 0.53), peer-reviewed publications (p = 0.97), relying on repetition/reproducibility (p = 0.10), and checking the information’s matching with their clinical findings (p = 0.87) to determine the reliability of information used in addressing the benefits and harms of treatments (Table 1).

We did not detect any statistically significant association between the providers’ experience level and their use of the reputation of the source (p = 0.89), traditional critical appraisal (p = 0.43), peer-reviewed publications (p = 0.84), relying on repetition/reproducibility (p = 0.29), and checking the information’s matching with their clinical findings (p = 0.41) to determine the reliability of information (Table 2).

Almost all the providers (113/124) reported ease of access to information that they seek to address the benefits and harms of treatments. We did not observe any variation in the ease of access to information across providers from various specialties (p = 0.41) (Table 1). Most providers were either satisfied (76/124) or somewhat satisfied (29/124) with their current process of seeking information to address the benefits and harms of treatments. Providers who reported being slightly satisfied requested access to more resources, remote access to library materials, and training on searching and critically appraising evidence. We did not notice any variation in the degree of satisfaction with seeking information across providers from various specialties (p = 0.30).

However, we found that providers who have recently finished their training (median experience = 6 years; range = 3-15) reported being not satisfied with their process of seeking information compared with providers with a greater level of experience (median experience = 15 years; range = 3-45) reporting being satisfied and (median experience = 14.5 years; range = 4-34) being somewhat satisfied (p = 0.04).

## Discussion

We report the information-seeking behaviors of 124 doctors from an American independent academic medical center hospital network. This cohort comprises approximately 10% of the network’s medical providers, which can be extrapolated to represent the entire membership. We found that most physicians across all medical specialties used and preferred point-of-care tools such as DynaMed and UpToDate, and many also used library websites to access journals and the PubMed database to search for information regarding the benefits and harms of treatments. The provider’s choices regarding these information sources appear independent of their experience level. It is important to note that many providers conduct a traditional critical appraisal. Still, many also rely on the reputation of the information source, such as a journal’s impact factor and the author’s research credentials, to determine the credibility and reliability of the information they seek regarding the benefits and harms of treatments. Providers who have recently completed their training are more likely to use the traditional critical appraisal to determine the credibility of information and seek improvements in related institutional resources than senior providers.

Nonetheless, most providers were satisfied with their current process of seeking information to address the benefits and harms of treatments. Most providers reported that their patients brought treatment and diagnosis-related information to the consultation. However, fewer emergency medicine providers said their patients brought information about the treatment compared with other specialties. Nonetheless, the number of patients bringing information to the consultation was similar across medical specialties. As expected, almost all patients searched the internet for information.

In contrast, only a few relied exclusively on other sources, such as family, friends, television commercials, and print media. Most providers, if not all, reported acceptance and tended to motivate the patients. Providers across specialties reported that their patients had specific questions about treatment benefits and costs, followed by particular questions regarding treatment-related adverse events.

Our findings, which showed that physicians prefer point-of-care tools such as DynaMed and UpToDate, were expected and consistent with other studies [7,11]. Overall, we did not notice significant variations in physicians’ information-seeking behaviors across various medical specialties. In contrast, Bennett et al. reported that family physicians were more likely than specialists to use handheld computers [7]. However, we found that younger physicians are more likely to use traditional critical appraisal to determine the credibility of information and seek improvements in related institutional resources. Bennett et al. reported that younger and female physicians were most likely to seek information [8]. We did not collect data on the physicians’ gender and could not conduct a similar analysis. However, it is essential to note that the physician’s experience level and related information-seeking behavior might not impact patient outcomes. Some studies have reported that physicians who have been in practice for longer periods may be at risk of providing lower-quality care [12,13]. However, we did not assess the physician’s experience level, related information-seeking behavior, or its relative impact on patient outcomes. Many studies have reported that health professionals feel threatened by patients’ information and respond defensively by asserting their “expert opinion” [14]. However, it is important to note that most physicians in our study reported that they demonstrated acceptance and tend to motivate their patients. The majority also mentioned that they have had informed discussions with their patients in the context of providing them with information. While this is undoubtedly a step toward shared decision-making, considerable work remains to be done.

The number of people turning to the internet to search for various health-related subjects continues to grow. In all, 80% of internet users, or about 93 million Americans, had searched for a health-related topic online. This is up from 62% of internet users who went online to research health topics in 2001 [14]. Overall, the number of patients bringing information to the consultation is up, and it may be comparable across specialties. In our study, the distribution of the number of patients seen per month and the average percentage of patients bringing information that they had searched for during the consultation were similar across medical specialties.

On the other hand, it is more and more certain that younger physicians are searching for information to stay current with advances in treatment and using the best and most up-to-date treatments for their patients, hoping for the best outcomes. Studies have reported that physician searches seemed to induce patient anxiety but more often led to patient comfort, clinical understanding, and enablement. Patients were more likely to report that web searches had a positive impact on the patient-clinician relationship. However, the nature of the effect may depend on the clinician’s response to the patient’s queries about the information. Physicians commonly perceived impartial effects on patients and the patient-clinician relationship, and frequently raised apprehensions about the correctness of online information [15]. However, in our study, physicians were receptive and had informed discussions with their patients.

We believe that healthcare providers should take a more active role in guiding patients to credible, evidence-based sources of health information, including web-based sources to complement consultation in the doctor’s office. Such a collaborative approach may lead to better shared decision-making, improving patient-clinician relationships and superior health outcomes. However, more robust research is needed, primarily through studies that investigate the accuracy and level of evidence of content searched and used by patients and clinicians. Future studies should also examine the impact of physicians’ information-seeking behaviors (across specialties and levels of experience, including those in solo practice versus group practice) and their effect on patient outcomes.

Some of the strengths of our study include employing a rigorous, cross-sectional, mixed-methods approach that combines structured one-on-one interviews, interviewer training and standardization, blinded transcription, dual independent hand-coding with consensus validation, and appropriate non-parametric analyses. By quantifying resource use, credibility and reliability heuristics, and user satisfaction, the study yields actionable targets for faculty development and institutional support to enhance point-of-care evidence use and shared decision-making. However, our study has some limitations, including being a single-center study from an independent academic medical center, which limits its external validity and may not generalize to other healthcare systems. The data are self-reported from structured one-on-one interviews, which introduces susceptibility to recall and social desirability biases, particularly because participants were informed of the study context and hypotheses.

## Conclusions

In this multi-department survey of 124 providers, most respondents reported that patients brought information about their treatment and diagnosis, with no significant variation across specialties in the topics presented by patients, including diagnosis, prognosis, and others. Providers predominantly relied on point-of-care tools, including DynaMed, UpToDate, and Lexicomp, and just over half used PubMed, while fewer consulted clinical practice guidelines, textbooks, or colleagues. Only one-third reported conducting traditional critical appraisal, and the specialty-level use of the library website or consultation with colleagues did not differ. Providers who had completed training more recently were more likely to be unsatisfied with their information-seeking process than their more experienced peers, who were satisfied or somewhat satisfied. Overall, physicians preferred point-of-care resources but often judged their credibility by the source’s reputation, indicating a need for targeted training to strengthen evidence appraisal at the point of care.

## Appendices

Interview questionnaire

1.      What year did you graduate from medical school?

2.      What year did you complete your last training (e.g., fellowship, etc.)?

3.      In which country is your medical school located?

4.      What is your specialty?

5.      What is the average number of patients you see every month?

6.      What is the average number of patients per month who bring along searched information during visits?

7.      When patients bring information during consultation, what is it about? Probe further if it is about treatment, diagnosis, prognosis, etc.?

8.      What are the typical sources of information patients generally bring during visits? Can you give an example?

9.      What is your reaction to patients bringing searched information during a consultation? Probe further, whether it is frustration, motivation, anger, etc.

10.  What response do you get from patients when you do not support their sources? Probe further, whether it is frustration, motivation, anger, etc.

11.  What kind of questions do patients typically ask about the benefits and harms of treatments?

12.  How much time, on average, do you spend discussing the information brought by the patients? Probe further if it is too time-consuming, or would it be better if more time were available, etc.?

13.  How do you search for information on benefits (e.g., survival, event-free survival, response rate, etc.) and harms (e.g., treatment-related mortality and morbidity) of treatments? (Probe: Do you go through goggle.com/or the LVHN library website, use DynaMed or similar software on your phone/tablet/computer, etc.?).

14.  Where do you search for information on the benefits and harms of treatments? (Probes: which database/books/journals/consult colleagues/explore professional society websites, etc.)

15.  How do you determine the credibility of the information you accessed on benefits, harms, and treatments?

16.  How do you define reliability?

17.  Do you have any preference for a certain information resource? Probe further if the preference for one resource is for answering a specific question (e.g., for benefits one resource and for harms another).

18.  Why do you prefer this (these) specific source(s)?

19.  Are you able to easily access the desired source?

20.  Do you prefer to use a certain device to access this information (such as a computer, phone, etc.)? Probe-based on office place (main hospital, Cedar Crest building, satellite office, etc.)

21.  Do you have the resources to access your desired source? Probe to see if their phone or computer can access subscription services or websites (such as DynaMed or UpToDate).

22.  How do you decide if the resources you accessed are sufficient to answer patients’ queries?

23.  Are you satisfied with your current process of seeking information on the benefits and harms of treatments?

24.  What kinds of faculty development sessions will be beneficial for you in addressing your evidence search needs?

25.  How often would the sessions need to be offered?

26.  What are your preferences for the location of these sessions?

27.  What else do you need to meet your needs better related to the evidence you tend to use for shared medical decision-making?

28.  Do you use software programs to assist you in decision-making at the patient’s bedside? (For example, do they use DynaMed or UpToDate?) Do you have any preference for a program? Ask about details regarding the subscription level: is it paid or free?

29.  Currently, how are these programs paid for? Do you pay out-of-pocket or use department continuing medical education (CME) funds for these services?

## References

[1] Eysenbach G (2003). The impact of the Internet on cancer outcomes. CA Cancer J Clin.

[2] Tang H, Ng JH (2006). Googling for a diagnosis--use of Google as a diagnostic aid: internet based study. BMJ.

[3] Edwards BK, Brown ML, Wingo PA (2005). Annual report to the nation on the status of cancer, 1975-2002, featuring population-based trends in cancer treatment. J Natl Cancer Inst.

[4] Helft PR, Hlubocky F, Daugherty CK (2003). American oncologists' views of internet use by cancer patients: a mail survey of American Society of Clinical Oncology members. J Clin Oncol.

[5] Charles C, Gafni A, Whelan T (1991). Decision-making in the physician-patient encounter: revisiting the shared treatment decision-making model. Soc Sci Med.

[6] Murray E, Pollack L, White M, Lo B (2007). Clinical decision-making: physicians' preferences and experiences. BMC Fam Pract.

[7] Bennett NL, Casebeer LL, Kristofco R, Collins BC (2005). Family physicians' information seeking behaviors: a survey comparison with other specialties. BMC Med Inform Decis Mak.

[8] Bennett NL, Casebeer LL, Zheng S, Kristofco R (2006). Information-seeking behaviors and reflective practice. J Contin Educ Health Prof.

[9] Casebeer L, Bennett N, Kristofco R, Carillo A, Centor R (2002). Physician Internet medical information seeking and on-line continuing education use patterns. J Contin Educ Health Prof.

[10] Mays N, Pope C (1995). Rigour and qualitative research. BMJ.

[11] De Leo G, LeRouge C, Ceriani C, Niederman F (2006). Websites most frequently used by physician for gathering medical information. AMIA Annu Symp Proc.

[12] Choudhry NK, Fletcher RH, Soumerai SB (2005). Systematic review: the relationship between clinical experience and quality of health care. Ann Intern Med.

[13] Tsugawa Y, Newhouse JP, Zaslavsky AM, Blumenthal DM, Jena AB (2017). Physician age and outcomes in elderly patients in hospital in the US: observational study. BMJ.

[14] (2025). NBC News. More people search for health online. http://www.nbcnews.com/id/3077086/t/more-people-search-health-online/.

[15] Wang J, Ashvetiya T, Quaye E, Parakh K, Martin SS (2018). Online health searches and their perceived effects on patients and patient-clinician relationships: asystematic review. Am J Med.

